# Infection, Dissemination, and Transmission Potential of North American *Culex quinquefasciatus*, *Culex tarsalis*, and *Culicoides sonorensis* for Oropouche Virus

**DOI:** 10.3390/v13020226

**Published:** 2021-02-02

**Authors:** Bethany L. McGregor, C. Roxanne Connelly, Joan L. Kenney

**Affiliations:** 1 Center for Grain and Animal Health Research, Agricultural Research Service, United States Department of Agriculture, Manhattan, KS 66502, USA; Bethany.McGregor@usda.gov; 2 Arboviral Diseases Branch, Division of Vector-Borne Diseases, Centers for Disease Control and Prevention, Fort Collins, CO 80521, USA; csz5@cdc.gov

**Keywords:** vector borne diseases, arboviruses, insect vectors, *Bunyaviridae* infections, Culicidae, Ceratopogonidae

## Abstract

Oropouche virus (OROV), a vector-borne *Orthobunyavirus* circulating in South and Central America, causes a febrile illness with high rates of morbidity but with no documented fatalities. Oropouche virus is transmitted by numerous vectors, including multiple genera of mosquitoes and *Culicoides* biting midges in South America. This study investigated the vector competence of three North American vectors, *Culex tarsalis, Culex quinquefasciatus*, and *Culicoides sonorensis*, for OROV. Cohorts of each species were fed an infectious blood meal containing 6.5 log_10_ PFU/mL OROV and incubated for 10 or 14 days. *Culex tarsalis* demonstrated infection (3.13%) but not dissemination or transmission potential at 10 days post infection (DPI). At 10 and 14 DPI, *Cx. quinquefasciatus* demonstrated 9.71% and 19.3% infection, 2.91% and 1.23% dissemination, and 0.97% and 0.82% transmission potential, respectively. *Culicoides sonorensis* demonstrated 86.63% infection, 83.14% dissemination, and 19.77% transmission potential at 14 DPI. Based on these data, *Cx. tarsalis* is unlikely to be a competent vector for OROV. *Culex quinquefasciatus* demonstrated infection, dissemination, and transmission potential, although at relatively low rates. *Culicoides sonorensis* demonstrated high infection and dissemination but may have a salivary gland barrier to the virus. These data have implications for the spread of OROV in the event of a North American introduction.

## 1. Introduction

Oropouche virus (OROV; *Bunyavirales*; *Peribunyaviridae*; *Orthobunyavirus*) is the causative agent of Oropouche fever, a febrile illness that occurs throughout South and Central America. Since its initial isolation in 1955 from a febrile patient in Trinidad and Tobago, OROV is likely to have affected more than half a million people [1]. Multiple large-scale outbreaks in countries like Brazil [2,3], Panama [4], and Peru [5] have been documented, as well as additional human cases and detections in sylvatic reservoir hosts throughout South America [6,7]. Currently, OROV infection is known to result in high levels of morbidity, however human fatalities are unknown from this virus [8]. The symptoms of OROV often closely resemble those of other febrile illnesses such as dengue and Zika. Therefore, it is likely that some cases of OROV have been previously misdiagnosed and this virus is more widespread than previously thought [4].

Oropouche virus transmission is maintained in two cycles. The first, an urban cycle, is maintained primarily by the biting midge *Culicoides paraensis* Goeldi and the mosquito *Culex quinquefasciatus* Say through humans [9]. The other is a highly complex sylvatic cycle involving numerous arthropod species, including *Aedes serratus* (Theobald)*, Cx. quinquefasciatus, Coquillettidia venezuelensis* (Theobald), and *Culicoides*, transmitting the virus amongst a variety of reservoir hosts, including birds, sloths, primates, and rodents [2,8]. Due to the great diversity of hosts and vectors associated with sylvatic transmission, it is likely that vectors and reservoirs in North America could be susceptible to OROV in an introduction event. North America is also home to numerous avian families that have susceptible South American members, such as the Tyrannidae (tyrant flycatchers), Troglodytidae (wrens), Fringillidae (finches), and Thraupidae (tanagers) [8,10]. Migration patterns of birds moving between North and South America could contribute to the potential movement and introduction of OROV, a phenomenon that has been speculated with the mosquito-borne West Nile virus [11] and with ticks carrying spotted fever *Rickettsia* agents and Lyme disease [12,13]. Flyway-mediated movement has also been proposed as one mechanism by which Eastern equine encephalitis has moved both within the United States [14] as well as into the United States from South America [15]

The lack of data on how OROV could establish and be transmitted in North America is concerning in light of other recent arboviral introduction events. Zika virus, which had been known for decades, presented with new, concerning symptoms in 2016 and created a public health crisis as the pathogen spread in southern Florida [16]. During 2005–2006, a single amino acid mutation in the Chikungunya virus genome led to a significant change in vector specificity leading to a major outbreak on Reunion island [17]. While OROV is not currently known to result in fatalities, orthobunyaviruses are known to undergo mutations at high rates leading to the evolution of new viral strains and sometimes completely novel viruses [18]. Recombination and reassortment of OROV with other orthobunyaviruses could also be a concern. Reassortment of OROV has already led to the establishment of numerous novel viruses including Madre de Dios and Iquitos viruses in South America [18,19]. Numerous orthobunyaviruses with human pathogenicity occur in North America, including Cache Valley virus, Jamestown Canyon virus, and La Crosse virus, which is concerning from a recombination perspective.

Vector control is often regarded as the primary method for controlling vector-borne disease outbreaks [20]. Increasing incursions of vector-borne diseases within the United States highlight our lack of preparedness for large-scale control of many vector species [21,22]. Furthermore, control strategies have not been developed for many North American *Culicoides* species, with most evaluations that have been conducted occurring in agricultural settings [23]. In the event of a North American OROV outbreak transmitted by a *Culicoides* species, there is currently no established large-scale control protocol for members of this genus. For these reasons, it is imperative that we gain a better understanding of the potential ecology of this pathogen prior to an introduction into North America. The present study investigated the vector competence of colony specimens of three common North American vector species including *Culex tarsalis* Coquillett, *Cx. quinquefasciatus*, and *Culicoides sonorensis* Wirth & Jones.

## 2. Materials and Methods

### 2.1. Insects

Two mosquito colonies were used for this experiment: *Culex tarsalis*, from the Bakersfield colony originally colonized from Bakersfield, CA, in 1952, and *Culex quinquefasciatus*, from the Sebring colony originally colonized from a population in Florida in 1988. Neither colony is outcrossed with wild members. Both *Cx. tarsalis* and *Cx. quinquefasciatus* were reared in the Centers for Disease Control and Prevention clean insectary in an incubator (Thermo Scientific, Thermo Fisher Scientific, Waltham, MA, USA) held at 27.5 °C on a 12:12 L/D schedule. *Culicoides sonorensis* individuals (from the Ausman colony established from a population collected in Brighton, CO, in 2001 that is not outcrossed with wild individuals) were received from the United States Department of Agriculture Arthropod-Borne Animal Diseases Research Unit (USDA ABADRU) as 1-day-old adults and held in incubators in the CDC clean insectary under the same conditions as mosquitoes. Adult mosquitoes and midges were provided with 10% sucrose solution *ad libitum* until infection. The day prior to infection, groups of ≤50 individuals for all three species were moved into 475 mL paper cups with double layered mesh on top. Cups of midges and mosquitoes were moved into the BSL-3 incubators for a 1-day acclimation period at 28 °C prior to blood-feeding. All adult mosquitoes and midges were between 3–7 days old for infections.

### 2.2. Infection

Midges and mosquitoes were fed 6.5 log_10_ plaque forming units (PFU)/mL of Oropouche virus. The viral strain used was derived from a 1955 viral isolate from a febrile patient in Trinidad and Tobago obtained from the CDC Arbovirus Reference Collection. The virus had a history of five passages in an unknown media, five passages in suckling mice, and one passage in Vero cells. The stock was passaged two additional times on Vero cells to a titer of 8 log PFU/mL prior to use in infectious blood meals. Virus in growth medium was diluted in defibrinated goose blood (Colorado Serum Company, Denver, CO, USA) for both *Culex* species and defibrinated calf blood (Colorado Serum Company) for *Culicoides.* Infectious blood meals were provided using a Hemotek blood feeding system (Hemotek Ltd., Blackburn, UK) with a Parafilm membrane for all species. Insects were provided access to blood heated to 37 °C in an incubator set at 28 °C for one hour prior to sorting. After blood feeding, mosquitoes were processed by exposing cups to −20 °C for 40 s and then sorting blood-fed mosquitoes in a petri dish on ice. Because midges could not be knocked down with cold due to susceptibility to water droplets, *Culicoides* were knocked down and sorted using a CO_2_ fly staging pad (Item #BGSU-12, Lab Scientific, Danvers, MA, USA). All blood engorged insects were placed into clean 475 mL paper cups in groups of ≤50 individuals and provided 10% sucrose solution ad libitum until collection at 10 or 14 days post infection (DPI). Additionally, a subset of individuals was fed clean blood without the addition of virus as controls for each cohort. The control individuals were exposed to the same knockdown and experimental conditions as the virus-fed individuals.

### 2.3. Collections

Insects were scheduled for dissection and saliva collection at 14 DPI. Poor survival by *Cx. tarsalis* individuals necessitated termination of their incubation period at 10 DPI. For this reason, a subset of *Cx. quinquefasciatus* were also collected at 10 DPI for a direct comparison with *Cx. tarsalis.* All *C. sonorensis* and the remaining *Cx. quinquefasciatus* were collected at 14 DPI. For each insect, legs and wings were collected into microcentrifuge tubes containing Dulbecco’s Modified Eagle Medium (Gibco, ThermoFisher Scientific, Waltham, MA) supplemented with 10% fetal bovine serum (Omega Scientific, Inc., Tarzana, CA, USA), 4% penicillin streptomycin (Gibco, Thermo Fisher Scientific), and 1% amphotericin b (Gibco, Thermo Fisher Scientific) (500 µL for mosquitoes, 300 µL for midges) with four 2 mm zirconium oxide beads (Glen Mills Inc., Clifton, NJ, USA). Saliva was collected by inserting the insect’s mouthparts into a 5 µL capacity capillary tube (Drummond Scientific, Broomall, PA, USA) partially filled with immersion oil to facilitate uptake of saliva [24]. Both midges and mosquitoes were allowed to salivate for one hour prior to collecting capillary tubes into supplemented media without beads (300 µL for mosquitoes, 100 µL for midges) and collecting bodies into a separate tube containing supplemented media (500 µL for mosquitoes, 300 µL for midges) and four 2 mm zirconium oxide beads.

### 2.4. Sample Testing

All samples were tested by plaque assay on Vero cells (African green monkey kidney cells). Body samples were tested first to investigate whether the virus was able to successfully infect midgut tissues and establish a midgut infection [25,26]. For those individuals showing positive body samples, legs were then tested to determine whether virus was able to escape the midgut and disseminate throughout the insect’s body. Finally, for those showing a disseminated infection in body tissues, saliva was then tested to determine whether the virus was able to infect the salivary glands and be transmitted in saliva. Body and leg samples were homogenized with a tissue lyser at 20 Hz for 6 min (Qiagen, Hilden, Germany) followed by centrifugation at 10,500 RPM for 5 min (Eppendorf, Hamburg, Germany). Saliva samples were centrifuged at 10,500 RPM for 5 min to extract all saliva from capillary tubes prior to plating. Serial dilutions in complete media were used to titrate samples as necessary. Due to the extremely small volume of saliva produced by *Culicoides,* samples that did not produce positive results by plaque assay were further tested by cytopathic effects assays in order to identify any virus positive saliva samples. Cytopathic effects assays were conducted by pipetting 50 µL of the sample and 50 µL complete media onto monolayers of Vero cells in 6-well plates. The plates were then rocked every 15 min for 1 h to facilitate viral entry into cells. An additional 2 mL complete media was then added to each well and plates were observed for up to 2 weeks for cytopathic effects.

### 2.5. Data Analysis

Infection, dissemination, and transmission rates were calculated for each species and time point studied. Infection rates were calculated as the number of positive bodies divided by the total number of individuals assayed. Dissemination rates were calculated as the total number of positive legs divided by the total number with positive bodies. Transmission potential rates were calculated as the total number of positive saliva samples divided by the total number with positive body samples. Overall infection, dissemination, and transmission potential rates were also calculated by dividing the number with positive bodies, legs, and saliva, respectively, by the total number that took an infectious blood meal for each cohort.

When adequate data were available, titers were evaluated for normality through data visualization and Shapiro–Wilk tests. The titer data were determined to be non-normally distributed (Shapiro–Wilk *p* < 0.001), so titers were directly compared through Mann–Whitney U tests. Fisher’s exact tests using Bonferroni corrections were then used to evaluate whether rates of infection, dissemination, and transmission potential were significantly different between study species and between 10 and 14 DPI results for *Cx. quinquefasciatus.* Finally, Mann–Whitney U tests were run to determine whether mortality observed in experimental cohorts was significantly different from control cohorts. All statistics were run using the *stats* package in R statistical computing software (R Core Team, Vienna, Austria) [27].

## 3. Results

### 3.1. Infection, Dissemination, and Transmission Potential

**Figure 1:**
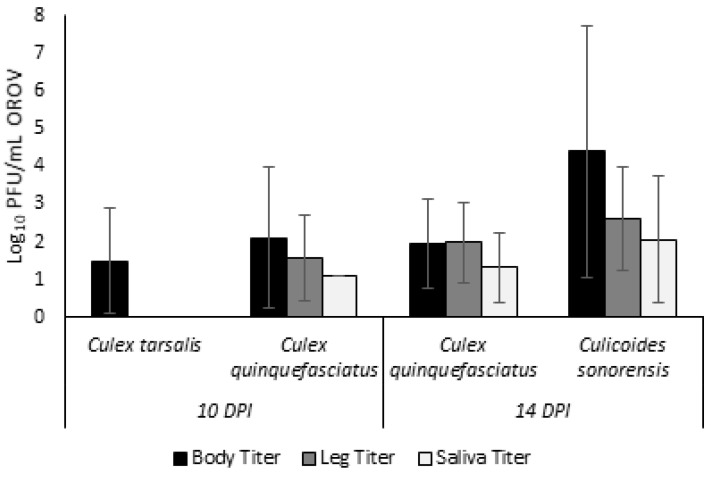
Viral OROV titers present in bodies, legs, and saliva of *Culex tarsalis* and *Culex quinquefasciatus* at 10 days post infection and *Culex quinquefasciatus* and *Culicoides sonorensis* at 14 days post infection. Titers are depicted as log plaque forming units per mL.

All *Cx. tarsalis* and a subset of *Cx. quinquefasciatus* were sampled on 10 DPI due to poor survival of *Cx. tarsalis* individuals. For *Cx. tarsalis*, 3 out of 96 individuals (3.13%; Supplementary File S1) had detectable virus in body samples (Table 1). The average viral titer for *Cx. tarsalis* bodies (*n* = 3) was 31.13 PFU/mL (Figure 1). There were no positive leg or saliva samples for *Cx. tarsalis*.

For the cohort of *Cx. quinquefasciatus* sampled at 10 DPI, 10 out of 103 individuals (9.71%, Table 1) had positive bodies at an average viral titer of 127.72 PFU/mL (Figure 1). Of the 10 *Cx. quinquefasciatus* with positive body samples, three (30%) showed evidence of a disseminated infection through positive plaque assays of leg tissues at an average viral titer of 36.65 PFU/mL. Finally, one individual (30%) had a positive salivary sample indicating transmission potential. However, the viral titer of this one sample was just 13.33 PFU/mL, likely a salivary titer too low for transmission to occur.

The remaining *Cx. quinquefasciatus* were sampled at 14 DPI. Out of 244 individuals assayed, 47 had positive body samples (19.3%, Table 1) with an average viral titer of 87.84 PFU/mL (Figure 1). Dissemination was observed for three of these 47 individuals (6.38%) with an average viral titer of 100 PFU/mL. Finally, two of these individuals (4.26%) with a disseminated infection further showed a transmissible infection with presence of virus in saliva samples. The average salivary titer for *Cx. quinquefasciatus* at 14 DPI was 21.67 PFU/mL.

All *C. sonorensis* individuals were collected at 14 DPI. Out of 172 total individuals assayed, 149 developed body infections (86.63%, Table 1) with an average viral titer of 2.5 × 10^4^ PFU/mL (Figure 1). Dissemination was observed in 143 of these individuals (95.97%) with an average leg titer of 404 PFU/mL. Saliva was assayed both through plaque assays and cytopathic effects assays. By plaque assay, 24 positive saliva samples were identified at an average salivary titer of 115.42 PFU/mL. Cytopathic effect assays identified an additional 10 positive samples bringing the total transmission potential rate to 34 of 149 individuals (22.82%).

### 3.2. Statistical Analyses of Viral Titers and Infections between Vectors

Due to the difference in incubation time, the *Cx. tarsalis* could only statistically be compared with the 10 DPI *Cx. quinquefasciatus*. While the average viral titers for *Cx. quinquefasciatus* bodies were higher than that of *Cx. tarsalis*, Mann–Whitney U tests determined this was not significant (W = 11, *p* = 0.54). No further statistical analyses of titer were completed for this pairing as no *Cx. tarsalis* displayed any dissemination or transmission potential. Infection rates for *Cx. quinquefasciatus* were higher than for *Cx. tarsalis*, although this was not significant (Fisher’s exact *p* = 0.08).

For the 14 DPI comparison between *Cx. quinquefasciatus* and *C. sonorensis*, *C. sonorensis* had a significantly higher viral titer in bodies (W = 183, *p* < 0.001) and legs (W = 51.5, *p* = 0.03), but not in saliva (W = 21.5, *p* = 0.84). Furthermore, in Fisher’s exact tests, the rates of infection (*p* < 0.001) and dissemination (*p* < 0.001) were significantly higher in *C. sonorensis.* Transmission potential was not significantly higher (Fisher’s exact *p* = 0.15) in *C. sonorensis* than in *Cx. quinquefasciatus*.

Analyses were conducted to investigate whether the rates of infection and dissemination as well as viral titers varied significantly between 10 and 14 DPI in *Culex quinquefasciatus*. Significantly more *Cx. quinquefasciatus* were infected on 14 DPI than 10 DPI (*p* = 0.03), although no significant difference was observed in dissemination rates (*p* = 0.07). There was no significant difference in viral titers between 10 DPI and 14 DPI in bodies (W = 224.5, *p* = 0.83) or legs (W = 0, *p* = 0.08). Low transmission potential overall for *Cx. quinquefasciatus* prevented further analysis of saliva results.

### 3.3. Survival Rates of Experimental and Control Insects

A subset of individuals from each cohort were fed clean blood without the addition of virus as controls. For *Cx. tarsalis*, 7/11 unfed controls (63%) survived until 10 DPI. Experimental *Cx. tarsalis* experienced 41.2% survival through 10 DPI (96/233 individuals). While the survival of the experimental *Cx. tarsalis* was low, this was not significantly different from the controls (Fisher’s exact *p* = 0.21). All of the *Cx. quinquefasciatus* controls survived through days 10 and 14 (25/25 individuals, 100%). Experimental *Cx. quinquefasciatus* experienced 92% survival through 10 DPI (103/112) which was not significantly different from controls (Fisher’s exact *p* = 0.21). The experimental *Cx. quinquefasciatus* maintained through 14 DPI experienced 74.9% survival (244/326 individuals), which was significantly lower than the control survival (Fisher’s exact *p* = 0.002). There were 13 *C. sonorensis* maintained as controls, with 7 (50%) surviving through 14 DPI. Out of 308 experimental *C. sonorensis*, 172 survived through the 14-day incubation period (55.8%), which was not significantly different from the control survival (Fisher’s exact *p* = 1).

## 4. Discussion

Numerous arthropod vectors have been implicated in the spread of Oropouche virus in South and Central America. The present study investigated the vector potential for North American populations of common vector species. Both *Culex* species show very limited potential as competent vectors for OROV. *Culex tarsalis*, a common species throughout the western United States and present in parts of southern Canada and northern Mexico [28,29], was an inefficient vector, only sustaining infection but not dissemination or transmission potential of OROV. *Culex quinquefasciatus*, a confirmed South American vector whose range extends north into the southern United States [28,29], showed very limited vector potential but was able to achieve infection, dissemination, and transmission potential for some individuals. *Culicoides sonorensis* is a species with a large North American range including most of the United States west of the Mississippi river as well as parts of the southeastern United States, the southern portion of the Canadian provinces of British Columbia, Alberta, and Ontario, and in numerous states of Mexico as far south as Guerrero [30,31,32]. This species is a confirmed vector of a variety of animal pathogens such as bluetongue virus [33], epizootic hemorrhagic disease virus [34], Schmallenberg virus [35], vesicular stomatitis virus [36], and African horse sickness virus [37]. Based on the results of the present study, we can confirm that this wide-ranging species is also a competent vector of a human pathogen and was found to have the highest vector competence for OROV of the three species tested. This species sustained high levels of infection and dissemination and moderate levels of transmission potential.

The levels of infection, dissemination, and transmission potential observed inform us on the barriers to transmission that may be active in each species. Four barriers to transmission are typically acknowledged with mosquito infection, namely the midgut infection barrier, midgut escape barrier, salivary gland infection barrier, and salivary gland escape barrier [38]. *Culex tarsalis*, with low infection and no dissemination or transmission potential, likely has a barrier to midgut infection preventing the virus from establishing. For those few individuals where an infection was established, a midgut escape barrier likely prevented any further movement of the virus out of the midgut. For *Cx. quinquefasciatus*, low infection and dissemination rates at both 10 and 14 DPI indicate that there are likely midgut infection and escape barriers active in this species as well. It is difficult to determine whether a salivary gland infection barrier is active in either of these *Culex* species due to the low number of individuals with disseminated infections. At 14 DPI, 2/3 individuals had virus positive saliva which may indicate a low-level salivary gland infection and escape barrier in *Cx. quinquefasciatus*. Finally, *C. sonorensis* seems to lack a midgut infection and escape barrier with high infection and dissemination rates. However, the relatively low transmission potential identified with this species suggests the presence of a salivary gland infection and escape barrier. While we can make inferences about the presence of these barriers based on the infection, dissemination, and transmission potential rates, the specific barriers were not further investigated and require additional research.

The low transmission potential identified in *C. sonorensis* could also be attributable to difficulties in collecting adequate saliva for the detection of virus from members of this genus [39]. One previous study identified that numerous false negatives were generated from both capillary assays and honey card salivary assays conducted with *C. sonorensis* infected with epizootic hemorrhagic disease virus [40]. It is therefore possible that by only collecting saliva using capillary assays, we are missing a proportion of the transmission competent individuals with our assays [41,42]. Challenges to collecting saliva have been bypassed in other studies by testing full heads and using those data as a surrogate for transmission potential [36]. For our purposes of not only determining vector competence but identifying which barriers to transmission were active, it was imperative to collect saliva to determine whether virus was able to bypass the salivary gland infection and escape barriers.

In the present study, *Culex quinquefasciatus* showed relatively low rates of infection and dissemination, indicating that this species may not be an efficient vector of OROV. These data agree with previously conducted experiments finding low transmission potential of South American populations of this species in laboratory infection studies [43,44]. In fact, there is evidence that extraordinarily high viral titers of ≥9.5 log_10_ SMLD_50_/mL are needed to infect South American *Cx. quinquefasciatus* in the laboratory [44]. *Culex quinquefasciatus* is still often cited as a likely urban vector of OROV, although not as significant as *Culicoides paraensis* [45]. Minimum infection rates of OROV in *Cx. quinquefasciatus* collected during an outbreak of febrile illness in Brazil indicated that 2.3 out of every 1000 specimens was likely to be infected [46]. It is possible that the low competence of this species is overcome by the extremely high abundance often seen in urban and suburban areas [47]. Vectorial capacity is a measure of the ability of a species to transmit a pathogen that takes into consideration both intrinsic characteristics like vector competence and extrinsic incubation period as well as extrinsic factors such as vector density, host use, and survival [48]. Based on this equation, the greater density of a vector can balance out a lower vector competence, meaning that even though *Cx. quinquefasciatus* has low vector competence, it could still act as a significant viral vector due to abundance.

It is unclear whether infection rates for *Cx. tarsalis* would increase if given an additional four days of incubation. Experimentally infected *Cx. tarsalis* individuals in the present study experienced very poor survival compared to control individuals, although this effect was not significant. This may indicate a deleterious effect of OROV on *Cx. tarsalis*, a phenomenon that, while uncommon, has been observed in other mosquito-borne pathogens [49]. For *Cx. quinquefasciatus* which, at 14 DPI, did experience significantly more mortality than controls, we also have to wonder whether some individuals succumbed to the virus. It is also possible that this mortality resulted from the high titer of virus provided to these mosquitoes and with a lower titer, mortality rates may have been lower. Individuals that died prior to the 10- or 14-day incubation period were not tested for virus to determine whether they had developed an infection that may have led to mortality. Testing those insects could have provided some clarity on whether the viral infection was to blame for the mortality seen and should be considered in future studies where high mortality in experimental cohorts is observed. Regardless, while it is possible that higher *Cx. tarsalis* infection rates would have been seen with increased survival and additional incubation time associated with a lower titer infection, a comparative cohort of *Cx. quinquefasciatus* displayed higher infection as well as dissemination and transmission potential already occurring at 10 DPI fed the same high infectious titer of OROV.

The viral titer of 6.5 log_10_PFU/mL used in this study is high, but we believe it is a relevant viral titer for this initial investigation. There are very little data on the circulating OROV viremia reached by non-human hosts in the sylvatic cycle. Most field studies do not collect wild vertebrates with circulating virus and, instead, investigate antibody prevalence. However, in experimentally infected mice, there is evidence that peak viral load in the plasma reaches 10^6.0^ TCID_50_/mL [1]. In humans, titers were found to be between 4.5 and 5.7 log_10_PFU/0.1 mL in one outbreak [10] and viremia up to 7.3 log_10_SMLD_50_ was recorded for one patient in another study [50]. Titers are highest during the first two days of illness followed by a decrease of one log on day three and a significant decline in following days [2], indicating that transmissible viremia probably does not last long in most patients. In one study conducted with the South American vector *Culicoides paraensis*, midges were successfully infected when feeding on human blood collected within the first two days of illness representing titers of ≥5.3 log_10_SMLD_50_ [50]. Circulating titers of ≤5.2 were inadequate to orally infect *C. paraensis*. Based on this information, the present infectious titer given of 6.5 log_10_PFU/mL should be within a reasonable viremia expected in a human host and represents a titer that can adequately infect another competent vector species.

The present study was meant to act as a pilot study for the evaluation of North American vectors for OROV, and the authors acknowledge limitations of the study design and utility. A major limitation is the age of the three colonies used for the infection assays. These assays utilized colonies that were available at the time of the study and the results should be interpreted as pilot data that can be used to guide future studies using younger colonies or low-generation field collected populations. However, based on the extremely low infection rates and the lack of evidence for dissemination or transmission, future studies with field populations of *Cx. tarsalis* are likely not warranted for OROV. The slightly higher competence of *Cx. quinquefasciatus* and its status as a confirmed vector in South America suggests this species would benefit from further studies. The high levels of infection and dissemination and moderate levels of transmission potential observed with *C. sonorensis* indicate that further studies with field populations are needed. Furthermore, it is unclear whether the use of two different blood sources (goose for both *Culex* species and calf for *C. sonorensis*) could have impacted the outcomes of these trials. The decision to use different blood sources was intended to increase blood feeding rates since each colony has been reared on their respective blood source over a long period. Future studies should endeavor to use more comparable blood sources when possible to eliminate this concern.

Another limitation of this study was the limited breadth of species evaluated. While the present study evaluated the vector competence of two different families of biting Diptera (Culicidae: *Culex*; and Ceratopogonidae: *Culicoides*), the known Culicidae vector breadth for OROV is much broader. Species such as *Aedes serratus* and *Ae. scapularis* (Rondani)*, Psorophora ferox* (Humboldt)*,* and *Cq. venezuelensis* have been implicated in the spread of OROV in South America [51]. For this study, *Cx. quinquefasciatus* and *Cx. tarsalis* were used because of accessibility of colony specimens and because of their significant role as arboviral vectors in North America. Additional studies will be needed to evaluate other common North American vector species, including species for which South American populations have been implicated. Furthermore, due to the role that avian migrations along flyways could potentially play, evaluating the vector competence of North American vectors that are known to feed on birds, such as *Culex pipiens* L. and *Culex restuans* Theobald [52], *Culiseta melanura* (Coquillett) [53], and *Coquillettidia perturbans* (Walker) [54], is especially warranted to determine the threat that flyway-mediated introduction poses.

The present study demonstrates different vector competence levels of three North American vectors for OROV, with *C. sonorensis* exhibiting the greatest competence. Numerous *Culicoides* species are present in North America [30], some of which are significant pests of humans such as the saltmarsh species *Culicoides furens* Poey. Although difficult to colonize, efforts should be made to evaluate the competence of other North American *Culicoides* species for OROV. Many North American *Culicoides* species are poorly studied, with numerous knowledge gaps pertaining to their ecology and control [23]. In the event of a *Culicoides*-driven OROV outbreak, control options to prevent widescale spread are limited and should be the focus of additional studies.

## Figures and Tables

**Table 1:** Overall rates of infection, dissemination, and transmission potential for *Culex tarsalis* and *Culex quinquefasciatus* at 10 days post infection and *Culex quinquefasciatus* and *Culicoides sonorensis* at 14 days post infection. Numbers in parentheses represent the percentage of individuals out of the total sample size tested.

DPI	Species	N	Infection	Dissemination	Transmission
10	*Culex tarsalis*	96	3 (3.13)	0 (0)	0 (0)
	*Culex quinquefasciatus*	103	10 (9.71)	3 (2.91)	1 (0.97)
14	*Culex quinquefasciatus*	244	47 (19.3)	3 (1.23)	2 (0.82)
	*Culicoides sonorensis*	172	149 (86.63)	143 (83.14)	34 (19.77)

## Data Availability

Data are available as Supplemental File S1: Experimental Data.

## Supplementary Materials

The following are available online at https://www.mdpi.com/1999-4915/13/2/226/s1, Supplementary File S1: Experimental Data.
